# Supplementary material for: Serotonin Transporter (SERT) Expression Modulates the Composition of the Western-Diet-Induced Microbiota in Aged Female Mice
Source: Nutrients. 2023 Jul 6;15(13):3048. doi: 10.3390/nu15133048 (PMC10346692; doi:10.3390/nu15133048)
Supplement: Supplementary file 1 [file nutrients-15-03048-s001.zip › nutrients-2470636-supplementary.pdf]

Supplementary Materials

Methods

Table S1. Number of faecal samples used per group and measurement point

	**Pre-intervention, Day 0 (T1)**		**Post-intervention, D 23 (T2)**	
**Diet**	**Control**	**Western**	**Control**	**Western**
Sert$^{+/+}$ **(WT)**	N=6	N=7	N=6	N=7
Sert$^{+/-}$ **(HET)**	N=7	N=8	N=7	N=8
Sert$^{-/-}$ (KO)	N=8	N=8	N=8	N=8

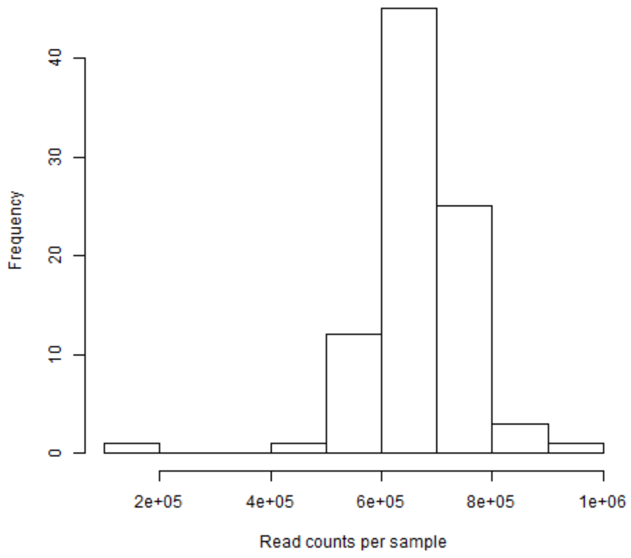

Figure S1. Histogram of raw read counts per sample.

Community analyses

Alpha-diversity:

Shannon alpha-diversity was estimated taking into account the amount of unique ASVs as well as their abundance. After assessing normality, repeated measures ANOVA was performed on the whole dataset using the within-subjects factor Time: Pre- intervention, day 0 (T1); Post- intervention, day 23 (T2) and the between-subjects factor Diet (Control diet, Western diet) and Genotype (WT, HET, KO) testing for effects of Time, Diet and Genotype. In case of significant global group differences on Genotype or interaction effects simple between group effects were assessed using *t-test*.

Beta-diversity:

As a measure of beta-diversity, Bray-Curtis dissimilarity was calculated using the function ordinate using the Principal Coordinate Analysis (PCoA) method. Bray-Curtis is a distance metric explaining differences in the relative abundances between microbial communities. Permutation testing was performed using the vegan function and adonis (Oksanen et al., 2019) on the whole dataset including all Diet, Genotype groups and Time points. Effects of Time: Pre- intervention, day 0 (T1); Post-intervention, day 23 (T2), Diet (Control diet, Western diet) and Genotype (WT, HET, KO) where assessed, accounting for repeated measures by using the 'strata' argument. In case of global group differences or interactions significant between group simple effects were assessed also using permutation testing.

Results

Community analyses

Alpha-diversity:

A main effect of Time was observed on Shannon diversity; $F(1,38)=94.71$, $p=.001$. Across Diet and Genotype groups the mean Shannon diversity decreased from mean (sd) 3.70 (.06) at T1 before the

dietary intervention to mean (sd) 2.72 (.08) after three weeks at T2. See **Figure S2** for the values of alpha-diversity per group. No further main or interaction effects of Genotype or Diet were observed.

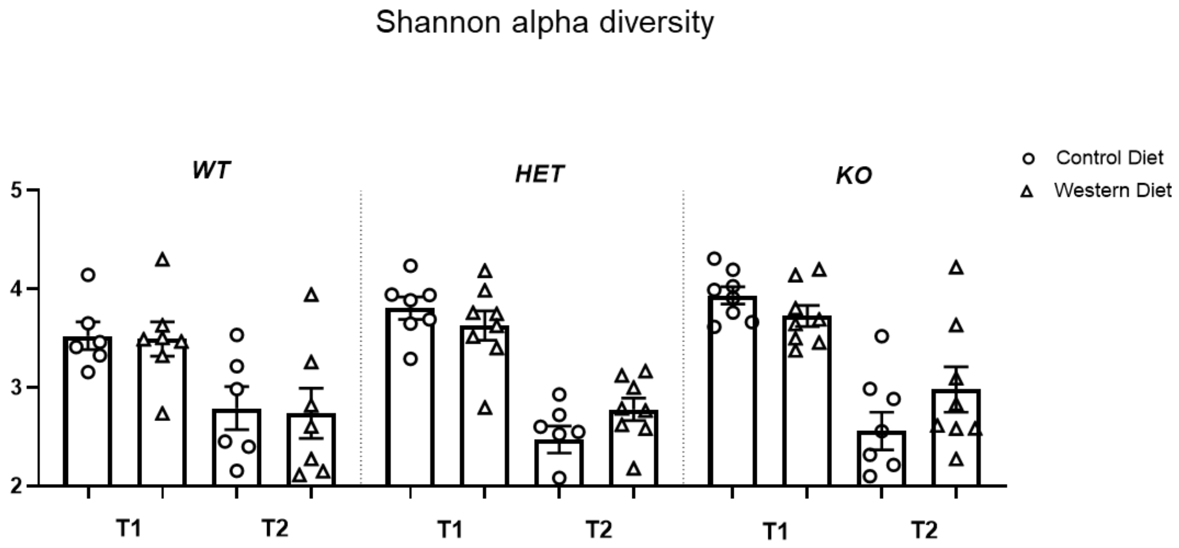

Figure S2. Boxplots of Shannon diversity for all groups. The dots are values higher or lower than 1.5 * the inter-quartile range. 'Control' – control diet, 'Western' – Western diet. T1 -Pre- intervention, day 0; T2- Post- intervention, day 23.

Beta-diversity:

Main effects of Time and Diet were found, as well as an interaction between Time and Diet (**Figure 3 left panel**) and an interaction between Time and Genotype (**Figure 3 right panel**), see **Table 3**. Gut bacterial communities of both Control and Western diet groups changed over time and, critically, did so differentially between Control and Western diet groups. Hence, Western diet differentially affected beta diversity compared to a Control diet.

There was also a differential change over time between the three genotype groups, irrespective of Diet indicating that the Western diet did not differentially affect SERT genotype groups. Further dissecting the Time points*Genotype interaction into simple effects shows that genotype effects were present but different at each time point; at T1, HET and KO groups were different, at T2, WT

and KO groups were different (see **Supplementary Table 2**). Within each Genotype group effects of Time are significant.

Table S2. Permanova results of Bray-Curtis beta diversity for the whole dataset (Time, Diet, Genotype main and interaction effects).

	**df**	**SumOfSqs**	**R2**	**Pseudo F**	**p-value**
Time	1	8.984	0.209	243.428	**0.001**
Diet	1	0.878	0.020	23.789	**0.008**
Genotype	2	1.044	0.024	14.140	0.054
Time*Diet	1	1.012	0.023	27.413	**0.004**
Main effect of Diet at T1	1	0.2152	0.025	1.0962	0.306
Main effect of Diet at T2	1	1.466	0.089	4.0893	**0.001**
Main effect of Time in the Western diet group	1	5.2207	0.289	17.859	**0.001**
Main effect of Time in the Control diet group	1	4.5811	0.293	16.553	**0.001**
Time points*Genotype (WT, HET, KO) #	2	1.199	0.028	16.245	**0.016**
Diet*Genotype	2	0.899	0.021	12.177	0.153
Time*Diet*Genotype	2	0.869	0.020	11.779	0.199
Residual	76	28.050	0.653		
Total	87	42.936	1.000		

Permanova results of Bray-Curtis beta diversity for the whole dataset (Time, Diet, Genotype main and interaction effects). The effects between the genotype groups are calculated in separate models and included the specified groups. Abbreviations: **df:** degrees of freedom, **SumofSqs**: sums of squares – average distances between groups, **R2**: variance explained, **Pseudo F**: signal to noise ratio of between to within group variance, p-value: fraction of permuted F ratio’s that are larger than the observed F ratio’s. # for simple effects dissecting the Time *Genotype interaction see below.

Table S3. Simple effects of Bray-Curtis dissimilarity between Genotype groups at the pre- and post-intervention Time points.

	WT T1	HET T1	KO T1	WT T2	HET T2
**HET T1**	.1350				
**KO T1**	.1671	**.0368**			
**WT T2**	**.0017**	**.0017**	**.0017**		
**HET T2**	**.0017**	**.0017**	**.0017**	.2080	
**KO T2**	**.0017**	**.0017**	**.0017**	**.0045**	.1671

Simple effects of Bray-Curtis dissimilarity between Genotype groups at T1 and T2 for three genotypes merged regardless diet used.

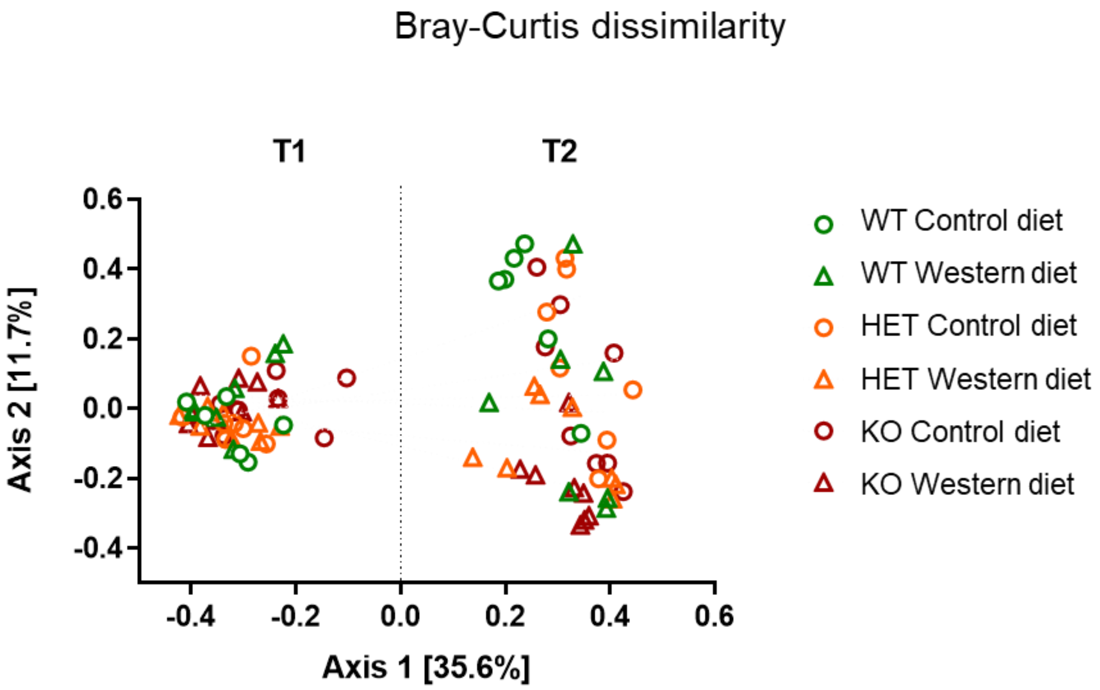

Figure S3. Two PCoA plots of Bray-Curtis dissimilarity metric for all groups, labelled to demonstrate significant effects of the permanova tests that are reported in Table 3. **Left panel**: T1 and Diet effects. **Right panel**: T1 and Diet effects and Genotype effects.

Taxonomy

Table S4. Median (range) of relative abundance per group (i.e. split by Time, Diet and Genotype group).

Genus	WT T1 Control	WT T1 Western	WT T2 Control	WT T2 Western	HET T1 Control	HET T1 Western	HET T2 Control	HET T2 Western	KO T1 Control	KO T1 Western	KO T2 Control	KO T2 Western
Carnobacteriaceae.Atopostipes	0 (0-0)	0 (0-0)	0 (0-0)	0 (0-0)	0 (0-0)	0 (0-0)	0 (0-0)	0 (0-0)	0 (0-0)	0 (0-0.001)	0 (0-0)	0.001 (0-0.009)
Ruminococcaceae.Intestinimonas	0.0395 (0.013-0.057)	0.047 (0.007-0.093)	0.016 (0.005-0.146)	0.044 (0.003-0.326)	0.036 (0.007-0.351)	0.027 (0.003-0.094)	0.024 (0.003-0.168)	0.06 (0.017-0.217)	0.116 (0.064-0.293)	0.0345 (0.005-0.126)	0.0825 (0.04-0.273)	0.2665 (0.129-1.221)
Erysipelotrichaceae.Erysipelatoclostridium	0.001 (0.001-0.203)	0.025 (0-0.119)	0 (0-0.013)	0.698 (0-6.43)	0.03 (0.009-0.363)	0.079 (0-0.668)	0 (0-0)	0.139 (0-4.312)	0.0065 (0.001-0.083)	0.003 (0-0.061)	0.1515 (0.007-0.283)	0 (0-0.001)
Ruminococcaceae.Ruminiclostridium.9	0.342 (0.238-0.674)	0.36 (0.159-0.945)	0.228 (0.074-0.560)	0.677 (0.178-1.591)	0.267 (0.08-0.471)	0.1825 (0.111-0.572)	0.203 (0.069-0.663)	0.4925 (0.079-1.659)	0.622 (0.264-1.949)	0.4415 (0.191-0.982)	0.398 (0.105-1.66)	0.835 (0.585-2.102)
Ruminococcaceae.Oscillibacter	0.2455 (0.137-0.507)	0.147 (0.062-1.553)	0.053 (0.026-0.15)	0.301 (0.092-0.711)	0.16 (0.021-0.3)	0.0815 (0.016-0.398)	0.109 (0.015-0.301)	0.3275 (0.023-0.84)	0.461 (0.176-1.21)	0.2655 (0.109-1.065)	0.1875 (0.073-1.216)	0.807 (0.355-1.279)
Ruminococcaceae.uncultured	0.158 (0.117-0.359)	0.267 (0.044-1.161)	0.097 (0.013-0.628)	0.17 (0.004-8.196)	0.214 (0.018-0.843)	0.09 (0.017-0.624)	0.025 (0.014-0.162)	0.35 (0.009-2.608)	0.6515 (0.222-3.984)	0.403 (0.072-0.927)	0.314 (0.052-1.259)	0.8075 (0.143-2.353)
Ruminococcaceae.Ruminiclostridium.5	0.163 (0.075-0.374)	0.192 (0.011-0.274)	0.061 (0.017-0.177)	0.22 (0.045-0.484)	0.157 (0.019-0.322)	0.118 (0.046-0.347)	0.061 (0.01-0.167)	0.1535 (0.009-0.444)	0.107 (0.072-0.316)	0.096 (0.017-0.25)	0.269 (0.042-0.524)	0.327 (0.202-0.663)
Ruminococcaceae..Eubacterium..coprostanoligenes.group	0.011 (0-0.1)	0.022 (0-0.235)	0.0745 (0.005-0.135)	0.254 (0.001-3.06)	0.105 (0.002-1.279)	0.0075 (0-0.112)	0.088 (0.008-0.471)	0.357 (0.001-4.487)	0.087 (0-0.295)	0.0215 (0-0.443)	0.026 (0-0.096)	1.3055 (0-10.529)
Lachnospiraceae.A2	0.05 (0.005-1.128)	0.08 (0.005-0.875)	0.4755 (0.159-2.531)	3.584 (0.249-11.331)	0.157 (0.075-0.939)	0.299 (0.011-0.785)	0.069 (0.007-0.277)	0.2865 (0.022-7.805)	0.23 (0.002-17.79)	0.223 (0.139-1.64)	0.26 (0.055-0.994)	0.238 (0.002-1.927)
Peptococcaceae.uncultured	0.098 (0.032-0.14)	0.04 (0.004-0.115)	0.026 (0.003-0.083)	0.105 (0.011-0.207)	0.101 (0.007-0.165)	0.07 (0.018-0.421)	0.066 (0.002-0.233)	0.0815 (0.001-0.273)	0.097 (0.039-0.341)	0.0485 (0.012-0.163)	0.117 (0.066-0.412)	0.2975 (0.057-0.641)
Streptococcaceae.Streptococcus	0.056 (0.028-0.085)	0.055 (0.013-0.079)	0.058 (0.031-0.61)	0.298 (0.043-0.766)	0.06 (0.008-0.108)	0.099 (0.051-0.105)	0.139 (0.025-0.34)	0.4525 (0.027-0.584)	0.0525 (0.006-0.134)	0.058 (0.007-0.163)	0.2945 (0.048-0.552)	0.5805 (0.326-1.044)
Staphylococcaceae.Staphylococcus	0 (0-0.002)	0.001 (0-0.001)	0.001 (0-0.021)	0.003 (0-0.292)	0.001 (0-0.002)	0.002 (0.001-0.016)	0.002 (0-0.003)	0.003 (0-0.065)	0.001 (0-0.004)	0.001 (0-0.002)	0.0035 (0-0.012)	0.019 (0.001-0.272)
Rikenellaceae.Alistipes	0.942 (0.491-2.987)	0.332 (0.302-1.654)	0.526 (0.046-3.535)	0.591 (0.043-3.159)	0.578 (0.141-0.936)	0.756 (0.366-2.351)	0.325 (0.095-0.641)	0.67 (0.229-5.207)	1.3835 (0.29-2.494)	0.8685 (0.595-1.484)	0.553 (0.225-1.257)	1.6625 (0.164-2.68)
Desulfovibrionaceae.Bilophila	0.004 (0-0.011)	0.016 (0.001-0.029)	0.0555 (0.020-0.330)	0.481 (0.078-2.191)	0.007 (0.001-0.027)	0.003 (0-0.014)	0.057 (0.01-0.176)	0.378 (0.023-1.647)	0.0065 (0-0.024)	0.0055 (0.002-0.14)	0.197 (0.037-0.76)	0.639 (0.169-2.687)
Erysipelotrichaceae.Faecalibaculum	0.058 (0.001-1.681)	0.065 (0.001-0.322)	14.7065 (2.321-48.2)	0.213 (0.006-21.929)	0.188 (0.031-0.98)	0.0425 (0-0.972)	12.91 (0.036-35.7)	0.0245 (0-3.193)	0.0855 (0.005-0.806)	0.4765 (0-4.897)	17.873 (0.01-46.042)	0.0215 (0-15.303)
Peptostreptococcaceae.Romboutsia	0 (0-0)	0 (0-0)	0.113 (0-2.631)	0.074 (0-0.201)	0 (0-0)	0 (0-0)	3.546 (0.044-5.123)	0 (0-0.881)	0 (0-0)	0 (0-0)	0.006 (0-7.78)	0.0215 (0-0.17)
RF39.uncultured.bacterium.uncultured.bacterium	0 (0-0.007)	0.004 (0-0.087)	0.001 (0-0.01)	0 (0-0)	0 (0-0.002)	0.0035 (0-0.086)	0.012 (0.002-0.049)	0 (0-0)	0.005 (0-0.021)	0 (0-0.064)	0.0025 (0-0.022)	0 (0-0.03)
Erysipelotrichaceae.Turicibacter	0 (0-0)	0 (0-0.176)	2.202 (0.07-10.23)	0.016 (0-0.284)	0.01 (0.006-0.872)	0.001 (0-0.005)	0.947 (0.091-6.477)	0.0345 (0-8.446)	0 (0-0)	0 (0-0)	0.009 (0-6.035)	0 (0-0.092)
Ruminococcaceae.Ruminococcaceae.UCG.014	0.2655 (0.07-0.565)	0.347 (0.029-1.876)	0.049 (0.003-0.211)	0.001 (0-0.356)	0.737 (0.112-4.452)	0.8135 (0.001-2.87)	1.103 (0.005-2.294)	0.0165 (0-0.152)	0.2375 (0.048-0.754)	0.2755 (0.015-2.938)	0.1065 (0.021-1.022)	0.009 (0-0.214)
Erysipelotrichaceae.Dubosiella	0.8025 (0.007-2.822)	1.975 (0-35.418)	3.79 (0.091-29.27)	0.008 (0.001-0.913)	0.701 (0.199-6.073)	0.2285 (0-2.548)	5.17 (0.005-16.11)	5e-04 (0-0.006)	0.0565 (0-4.243)	0 (0-15.042)	0.508 (0.005-28.06)	0 (0-0.001)

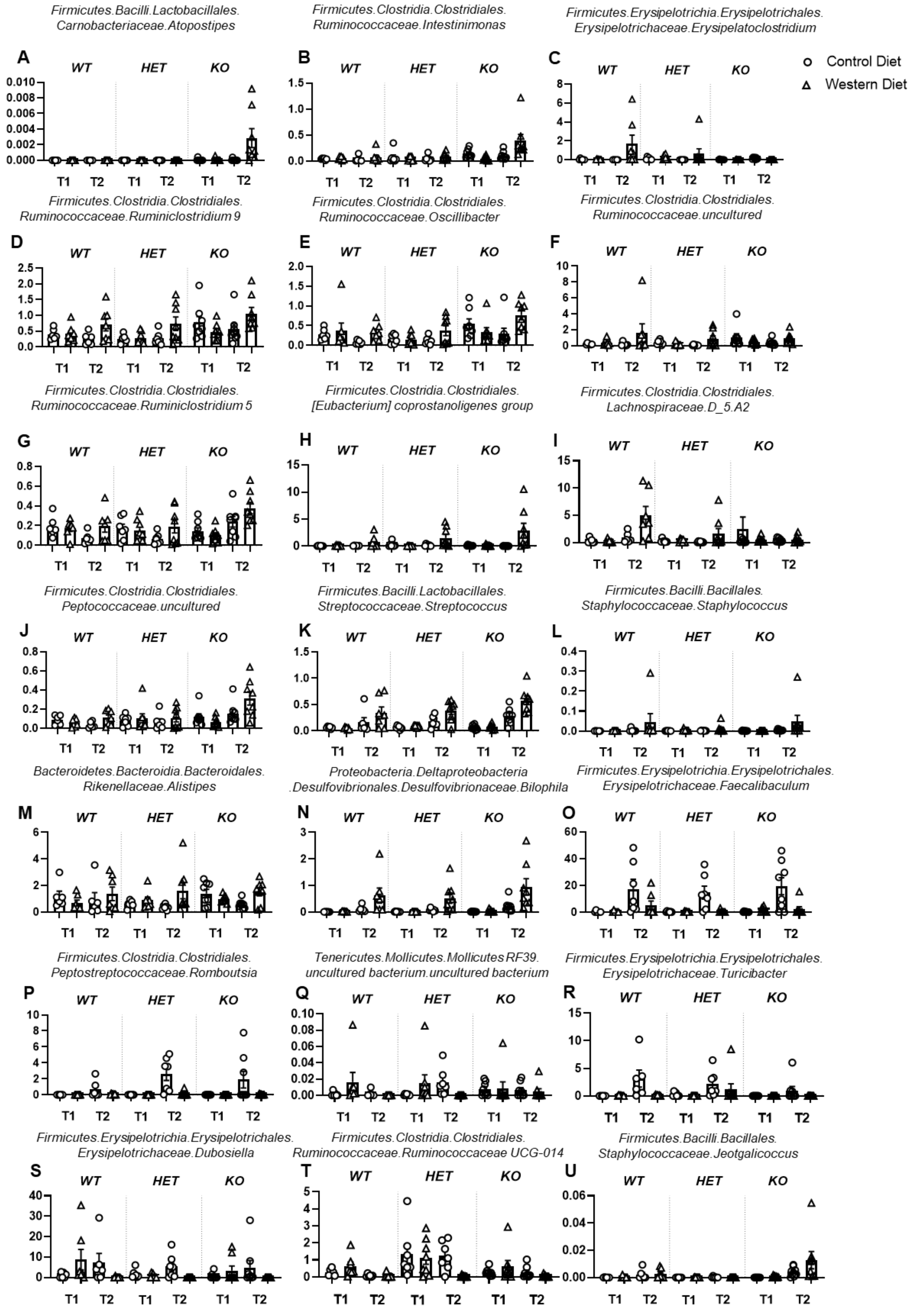

Figure S4. Boxplots of all groups for genera in which a main effect of Diet effect was observed (see **Table 1** main text).

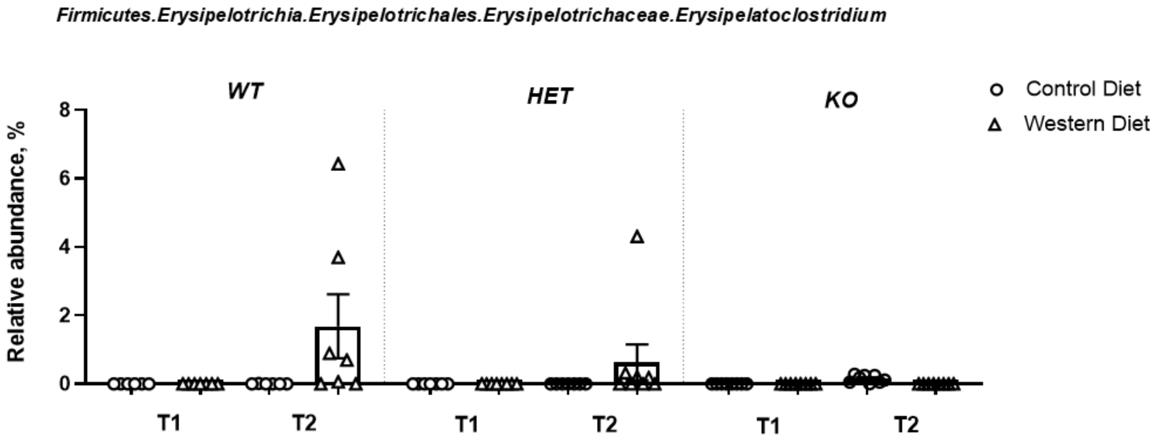

Figure S5. Effect of Diet and Genotype in the most abundant gera out of the five within genus *Erysipelatoclostridium.*
